# Hemothorax Following Traumatic Dobhoff Tube Insertion

**DOI:** 10.7759/cureus.13097

**Published:** 2021-02-03

**Authors:** Lindsey M Shain, Leslie McHale, Taha Ahmed

**Affiliations:** 1 Internal Medicine, University of Kentucky, Lexington, USA

**Keywords:** hemothorax, dobhoff tube

## Abstract

Dobhoff tube is a specialized small-bore and flexible nasogastric tube that makes it more comfortable for placement than a usual nasogastric tube. Dobhoff tube insertion is commonly considered a relatively safe bedside procedure, but it is not without its associated risks. Inadvertent tracheobronchial placement of Dobhoff tube has been associated with severe complications, most notably pneumothorax. We present a rare cause of right-sided hemothorax following tracheobronchial insertion of a Dobhoff tube with a prolonged and arduous clinical course.

## Introduction

Dobhoff and other flexible, small-bore nasogastric tubes are frequently placed for maintenance of enteral nutrition and delivery of medications in the inpatient setting. Dobhoff tube placement is typically considered a benign procedure and is most often performed using only visual and tactile clues to guide insertion during swallowing. Proper intra-gastric positioning is confirmed afterward via radiography [1,2]. A large majority of these cases are met with no complications. However, there have been several reported instances of inadvertent bronchopulmonary placement that have resulted in significant pulmonary trauma, particularly pneumothorax [3-8]. Hemothorax (a collection of blood within the pleural cavity) has been mentioned as a potential complication of a misplaced enteral feeding tube, but very few cases have been documented previously [9,10]. The aim of this report is to present a unique case of traumatic hemothorax caused by Dobhoff tube misplacement.

## Case presentation

**Figure 1:**
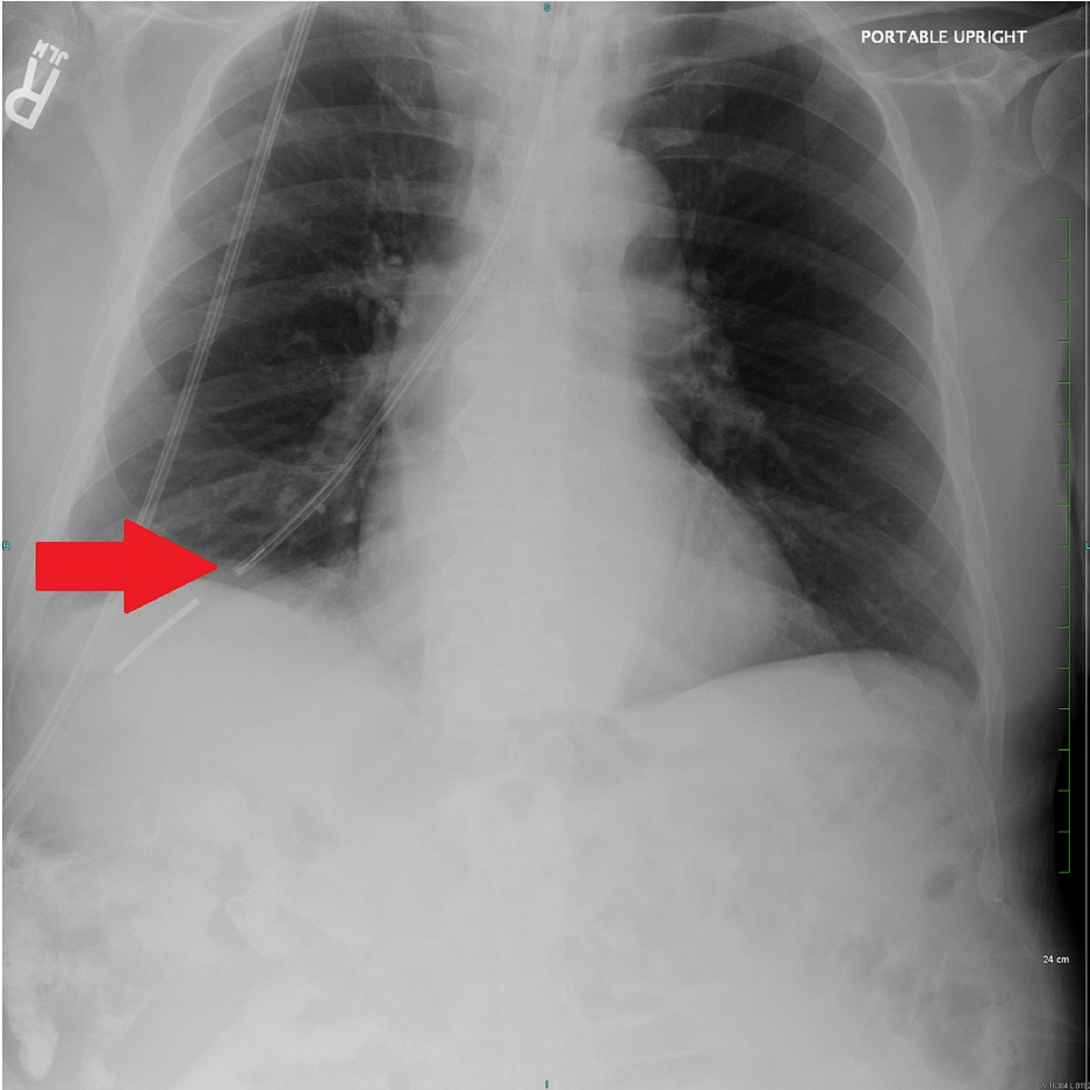
Chest X-ray demonstrating the Dobhoff tube penetrating the right lung base via the right mainstem bronchus (red arrow).

An 86-year-old Caucasian male with a history of hypertension, peripheral vascular disease, coronary artery disease, and rheumatoid arthritis presented to our facility as a direct transfer from an outside hospital (OSH) due to hemothorax. Per OSH documentation, the patient initially presented for acute encephalopathy. Due to his altered mental status, reported difficulties with swallowing, and concern for malnutrition, he was evaluated with a modified barium swallow study on hospital day (HD) 8. It demonstrated a high risk for aspiration, and a Dobhoff tube was subsequently placed at the bedside for delivery of enteral nutrition. A chest X-ray performed shortly after the tube placement demonstrated that the tip of the Dobhoff tube was within the right lung base, following the course of the right mainstem bronchus (Figure 1).

**Figure 2:**
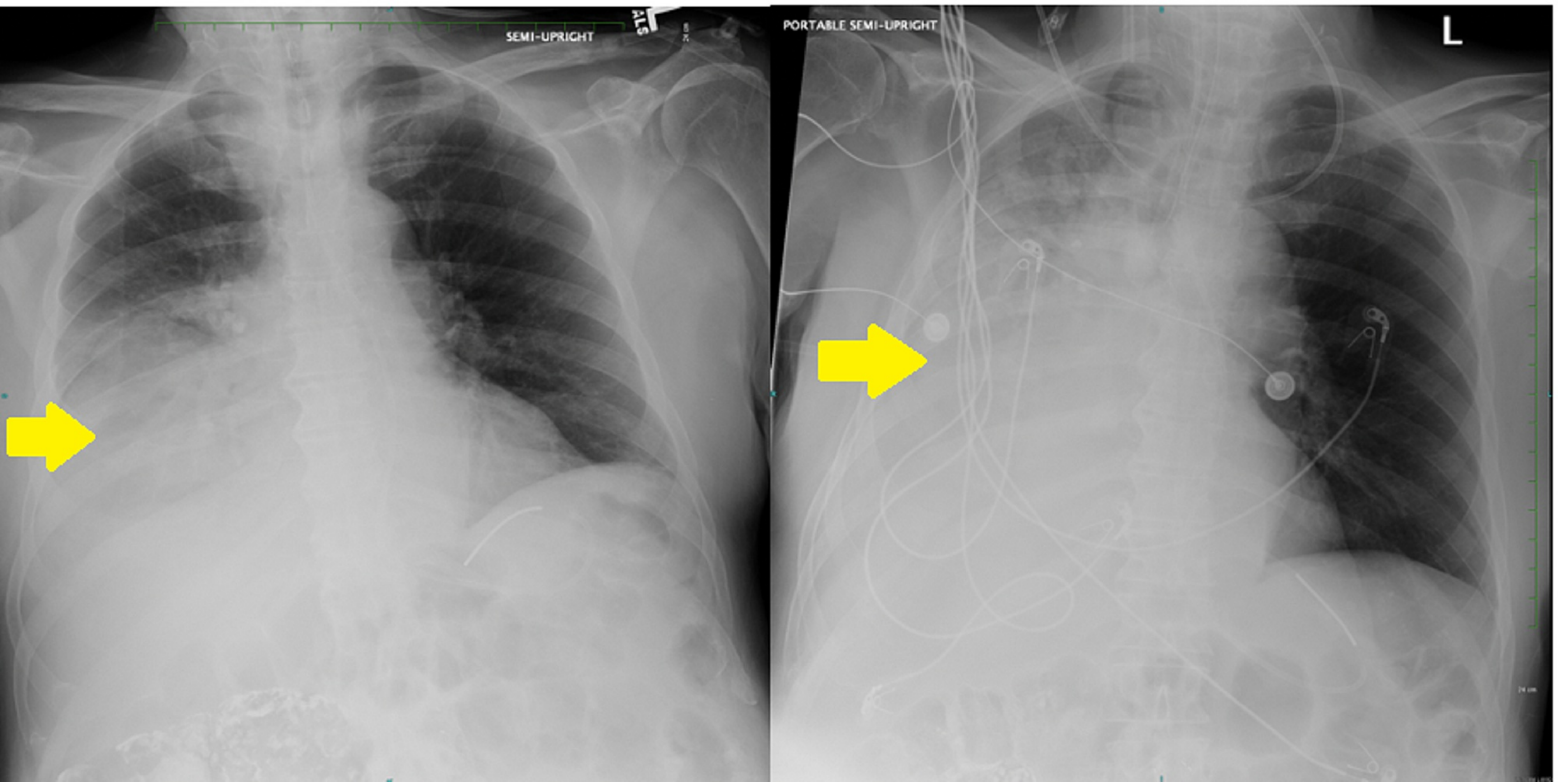
Sequential chest X-rays demonstrating progressive accumulation of right-sided pleural effusion (yellow arrows).

The Dobhoff tube was immediately removed and replaced on HD 9 under fluoroscopic guidance. Sequential chest X-rays on HD 10 demonstrated progressively increasing right-sided pleural effusion without evidence of pneumothorax (Figure 2).

The patient became hypoxic and was intubated for acute hypoxic respiratory failure. Hemoptysis was later observed on endotracheal tube suctioning. Chest computed tomography (CT) the following day revealed a large right-sided pleural effusion with areas of attenuation concerning for hemothorax, a right consolidating pneumonia, and a cavitating infiltrate in the right upper lobe concerning aspiration pneumonia. Broad-spectrum antibiotics were initiated, and a chest tube was placed for drainage of hemothorax.

On HD 17, the patient was transferred to our facility for continued intensive care unit (ICU) management. He arrived on mechanical ventilation, with a right-sided chest tube in place. Broad-spectrum antibiotic treatment was continued due to persistent right consolidation and right upper lobe cavitating infiltrate. During the ICU admission, the patient’s chest tube was replaced and intrapleural combination tissue plasminogen activator (TPA) and dornase were administered with complete resolution of the hemothorax. The patient was extubated on HD 33. Subsequently, the patient failed multiple modified barium swallow studies. Multiple Dobhoff tubes were replaced due to concern for aspiration, the necessity of maintaining enteral nutrition, and repeated removal by the patient. These were met with no further complications. The patient was ultimately given a percutaneous endoscopic gastrotomy tube with plans for discharge to a subacute rehabilitation center.

## Discussion

Small-bore nasogastric feeding tubes continue to be used widely to provide adequate enteral nutrition to patients with barriers to normal oral intake. Due to a typical blind placement procedure, abnormal positioning is often only appreciated after significant trauma is already dealt with in the lungs or other organs. Over the past few decades, the incidence of feeding tube misplacement into the bronchopulmonary system has remained around 1-3% of all tube placements, with pneumothorax being the most frequent complication [11,12]. Our case exemplifies another potentially life-threatening consequence of Dobhoff tube misplacement, hemothorax. Hemothorax from any cause is associated with a number of serious consequences such as atelectasis, anemia, respiratory failure, hemorrhagic shock, and infection of pleural fluid (i.e., empyema), among others [10]. In our patient, such dire outcomes were fortunately averted. Full resolution of the hemothorax was achieved with chest tube placement and treatment with TPA/dornase alpha.

Prior reports of traumatic Dobhoff placement have described situations in which attempts at insertion were met with some resistance [7]. Others described uneventful placements with no resistance or other hints that misplacement had occurred [4,10]. Because the inciting event in the current case occurred at a hospital outside of the authors’ institution, the full context surrounding the feeding tube misplacement is unclear. Regardless, clinical signs during blind placement are often unreliable indicators of proper positioning. Post-insertion X-ray remains the gold standard for placement confirmation but does not necessarily prevent adverse outcomes [12]. Newer techniques such as electromagnetic guidance allow for real-time tracking as a feeding tube is being advanced at a lower cost than traditional fluoroscopy and without exposure to ionizing radiation. This method has demonstrated a high success rate with reduced complications from pulmonary placement [13,14].

In general, we aim to highlight a severe complication that may arise from a routine procedure and to hopefully increase awareness of potentially fatal consequences. A growing body of evidence supports the safety, efficacy, and feasibility of new techniques for bedside guidance of Dobhoff tube insertion. Currently, fluoroscopy-guided placement in every patient is not a feasible solution due to cost and increased exposure to ionizing radiation. Additionally, it may take a few years for electromagnetic guidance to become more standard. Importantly, healthcare providers should be mindful of the potential risks when inserting Dobhoff tubes, and placement should at the very least be confirmed radiographically before use.

## Conclusions

Dobhoff feeding tubes are frequently placed by various healthcare providers to maintain enteral nutrition. Despite being perceived as a common and harmless procedure, feeding tube insertion is not without risk. Due to blind placement, Dobhoff tubes may be inadvertently positioned within the lung via the tracheobronchial tree, resulting in pneumothorax or hemothorax. This case report contributes to the literature by describing another rare instance of hemothorax from traumatic Dobhoff misplacement. Dobhoff placement should be confirmed radiographically in all cases. Additionally, the risk of misplacement may be mitigated by increased awareness of possible complications as well as the use of techniques for more accurate bedside guidance.
